# A Unique Case of Internuclear Ophthalmoplegia and Artery of Percheron Infarct

**DOI:** 10.7759/cureus.74744

**Published:** 2024-11-29

**Authors:** Liam D Redden, Gordon J Gubitz

**Affiliations:** 1 Medicine, Dalhousie University, Halifax, CAN; 2 Neurology, Dalhousie University, Halifax, CAN

**Keywords:** artery of percheron infarct, bilateral thalamic infarction, brain infarct, internuclear ophthalmoplegia, stroke

## Abstract

This case report discusses a unique presentation of an artery of Percheron (AOP) infarct resulting in rapidly resolving internuclear ophthalmoplegia (INO) without classical signs.

This is the case of a 70-year-old male patient who presented to a community Emergency Department following acute code stroke activation. Physical exam and imaging studies including non-contrast CT, CT angiography, CT perfusion, and MRI were performed. A review of the literature was also conducted.

MRI confirmed acute bilateral thalamic ischemic changes consistent with AOP infarcts. Symptoms of acute blurred vision, slurred speech, right hemi-face and tongue numbness, and signs of anisocoria, left INO, mild left skew deviation, and impaired convergence on the left resolved within 48 hours without intervention. The patient was discharged as neurologically normal and follow-up investigations did not identify a cause for the stroke.

The literature suggests varying presentations of AOP infarcts, with INO being rare. This case deviates from classical AOP presentations, emphasizing the importance of considering AOP infarcts early in differential diagnoses, particularly with unusual neuro-ophthalmological findings. Imaging modalities such as MRI with DWI prove crucial for early diagnosis, although AOP occlusions remain challenging to detect due to the small vessel size. Our case contributes to expanding knowledge of AOP infarct presentations and underscores the need for vigilance in recognizing atypical manifestations for timely diagnosis/management.

## Introduction

The artery of Percheron (AOP) is an anatomical variant of the P1 segment of the posterior cerebral artery (PCA) supplying the paramedian thalami and rostral midbrain [1]. The AOP variant is rare and estimated to be present in 4-12% of the population [2]. Rarer still are AOP ischemic strokes, which account for 0.1-2% of cerebral infarcts [3].

Clinical presentation of AOP infarcts is variable but classically involves a triad of altered consciousness, memory deficits, and supranuclear vertical gaze palsy. Other less common features include aphasia, dysarthria, focal motor weakness, cerebellar signs, parkinsonism, seizures, and hyperthermia [1].

We report a unique case of isolated neuro-ophthalmological deficits induced by an AOP infarct lacking the expected altered consciousness and memory deficits, which resolved quickly without acute intervention.

## Case presentation

A 70-year-old male presented to a community Emergency Department 30 minutes following the onset of acute ‘blurred vision’ with slurred speech and numbness of right hemi-face and tongue. ‘Code stroke’ was activated and he was transported emergently to the local stroke center. The slurred speech and sensory symptoms had resolved en route. 

On examination, he was alert and oriented with a Glasgow Coma Scale (GCS) of 15. Vital signs were within normal limits. Neurological examination revealed anisocoria with the left pupil (4mm) larger than the right (3mm). He had left internuclear ophthalmoplegia (INO) and endorsed diplopia on the right gaze and slight vertical diplopia in the primary position. He had mild left skew deviation and impaired convergence on the left. No nystagmus was noted. The remainder of the neurological examination was normal. 

Past medical history included previous right occipital lobe stroke treated with tenecteplase six months prior to the current presentation. His medications included clopidogrel (stroke risk reduction), rosuvastatin (dyslipidemia), and atenolol (hypertension).

A non-contrast computed tomography (CT) head scan, CT angiography, and CT perfusion study were performed as part of the acute stroke protocol. CT scan identified an old infarct in the right occipital lobe; no acute infarct was noted in the CT perfusion study. CT angiography (CTA) demonstrated calcific atherosclerosis of the carotid siphons and bilateral V4 vertebral arteries with no significant flow-limiting stenosis. 

After discussion with the patient, and with his symptoms rapidly resolving, decision was made not to treat with thrombolysis. He was admitted to the hospital with a suspected small brainstem stroke.

**Figure 1:**
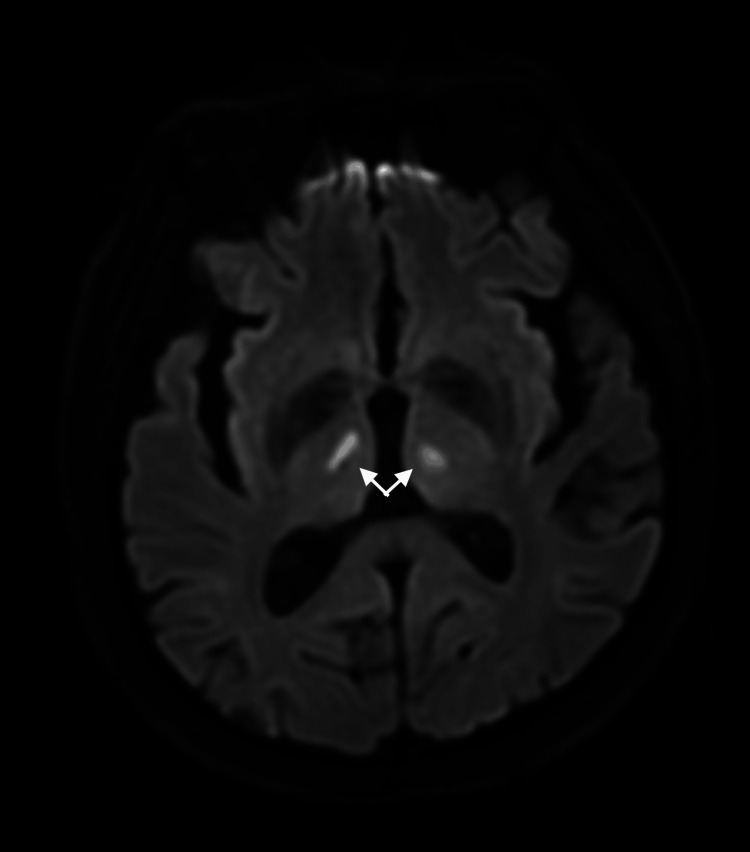
MRI-DWI sequence demonstrating bilateral thalamic ischemic strokes, indicated by white arrows. MRI-DWI: Magnetic resonance imaging-diffusion-weighted imaging

Magnetic resonance imaging (MRI) brain scan was performed the following day; axial T2 fluid-attenuated-inversion-recovery (FLAIR), diffusion-weighted imaging (DWI)/apparent diffusion coefficient (ADC), and gradient echo (GRE) sequences were obtained. The DWI sequence showed small acute bilateral thalamic ischemic changes in keeping with AOP infarcts, with corresponding restriction on the ADC sequence (Figure 1). 

**Figure 2:**
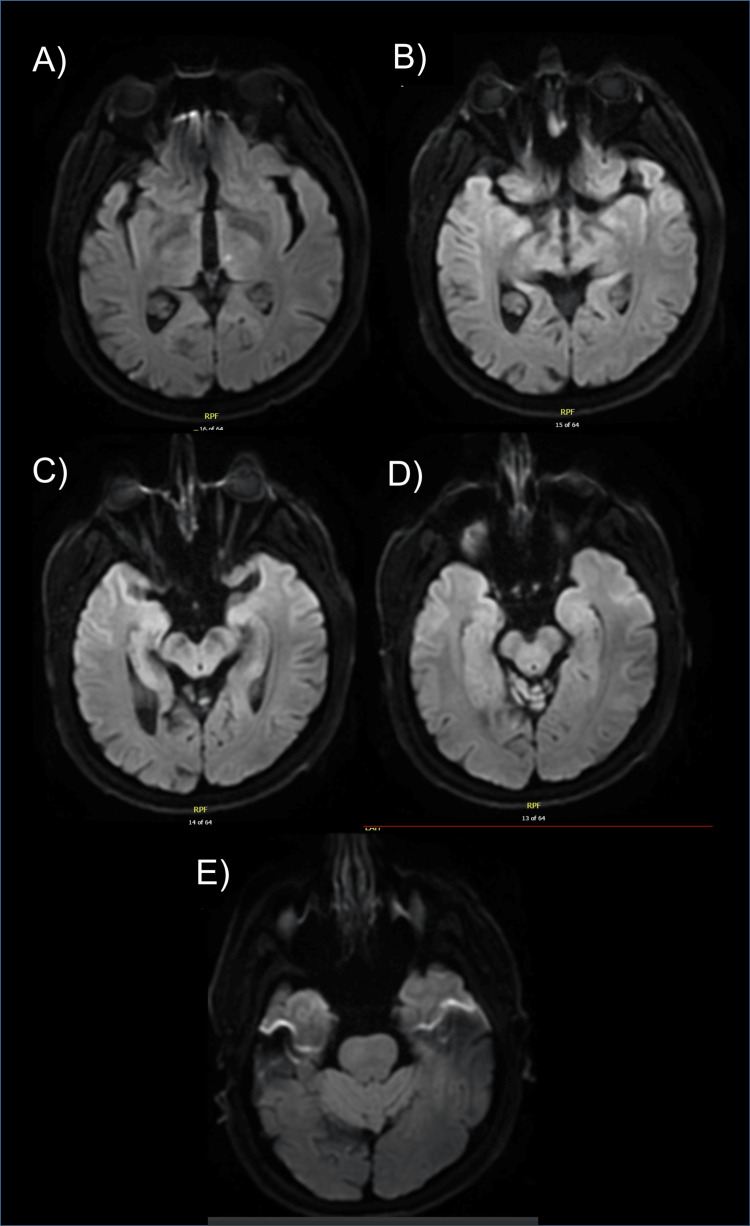
Axial DWI MRI images of midbrain and pons in order from most cranial (A) to most caudal (E). DWI: Diffusion-weighted imaging

Further DWI sequences demonstrating the status of the midbrain and pons are shown in Figure 2.

The previously noted right PCA territory infarct was seen, as well as generalized mild chronic small vessel disease and age-appropriate atrophy. 

Within 48 hours, his diplopia had completely resolved and his extraocular movements had returned to normal, apart from a slight asymptomatic reduction in left-side convergence. 

He was discharged home with appropriate secondary prevention strategies in place; urgent outpatient echocardiography/Holter monitoring was normal.

## Discussion

The three main symptoms of AOP infarctions described in the literature are vertical gaze palsy (65%), confusion/memory impairment (53 - 58%), and coma (42%) [4]. AOP infarctions should be kept on the differential with these presentations. Our case of transient speech and sensory disturbance and rapidly resolving neuro-ophthalmological findings (including an INO) was inconsistent with a ‘classical’ AOP presentation; AOP infarction was not on our differential, with the presentation more in keeping with brainstem pathology.

INO has been well described in the literature and can be caused by a brainstem lesion of any type that involves the medial longitudinal fasciculus (MLF) [5]. The classic presentation is impaired adduction ipsilateral to the lesion and abduction nystagmus (not always present) contralateral to the lesion with/without convergence interruptions [5].

Unilateral INO is sometimes associated with skew deviation/trochlear nerve palsy [5]. Skew deviation is probably due to interruption of otolithic projections that ascend within the MLF to the trochlear nucleus, oculomotor nucleus, and interstitial nucleus of Cajal [5].

Aladdin et al. published a case of vertical one-and-a-half syndrome as a result of AOP infarct [6]. In their case, vertical eye movements were limited particularly on upgaze bilaterally, attributed to partial damage of the oculomotor nuclear complex and the crossing fibers arising from the contralateral superior rectus subnucleus [6]. Our patient did not have vertical restrictions apart from a very mild vertical skew deviation noted on provocative testing, suggesting these structures had remained largely intact.

A larger case series described four main patterns of AOP infarcts [2]. These include bilateral paramedian thalami and rostral midbrain (43%), most in keeping with our case, bilateral paramedian thalami without midbrain involvement (38%), bilateral paramedian and anterior thalamic infarction with midbrain involvement (14%), and bilateral paramedian and anterior thalamic infarction without midbrain involvement (5%) [2]. Lazzaro et al. describe a distinctive imaging finding known as the midbrain ‘V sign’ with 67% sensitivity in cases of AOP infarction with midbrain involvement [2].

In our case, MRI showed some midbrain involvement which likely accounts for the MLF interruption. Puri and Sijapati reported a case of bilateral internuclear and internal ophthalmoplegia due to the AOP infarct and stated that due to the close physical proximity of the pair of white matter tracts that constitute the MLF at the periaqueductal gray in the midbrain, bilateral injury is common [7]. Symptoms in our case may be best explained by a rare unilateral injury in this area which quickly resolved. 

Multiple imaging modalities were required to ultimately secure the diagnosis. Kichloo et al. suggest in their review that CT, CT perfusion, CTA, and MRI (DWI and FLAIR) may be necessary depending on the clinical picture/available resources [1]. They proposed that MRI DWI and FLAIR were most appropriate for early diagnosis [1]. Proper diagnosis of AOP occlusion remains a challenge as the small vessel size makes the finding rare in conventional imaging modalities, including digital angiography [1].

To our knowledge, this is the first case report to describe an isolated left INO, slight left-sided vertical skew, and slight pupil size discrepancy as a potential result of AOP infarction. There are some limitations in our case to note. We did not measure saccadic velocities and gaze dependency of the INO was not determined at the time of presentation. In addition, although MRI demonstrated AOP infarction, it was not performed at the same time when symptoms were present at their peak severity. Therefore, it is possible that there was ischemia in other perforator branches caused by the top of the basilar embolization that was missed on MRI and that the severity of the ischemia was mild, leading to rapid relief of symptoms.

Our patient ultimately did well with symptom resolution within 48 hours of onset. No apparent cause for infarction was identified. While rare, this case adds to the growing literature on potential presentations of AOP infarcts.

## Conclusions

The literature suggests varying presentations of AOP infarcts, with INO being rare. This case deviates from classical AOP presentations, emphasizing the importance of considering AOP infarcts early in differential diagnoses, particularly with unusual neuro-ophthalmological findings. Imaging modalities such as MRI with DWI prove crucial for early diagnosis, although AOP occlusions remain challenging to detect due to the small vessel size. 
